# The genome-wide supported *CACNA1C* gene polymorphisms and the risk of schizophrenia: an updated meta-analysis

**DOI:** 10.1186/s12881-020-01084-0

**Published:** 2020-08-08

**Authors:** Yong-ping Liu, Xue Wu, Xi Xia, Jun Yao, Bao-jie Wang

**Affiliations:** School of Forensic Medicine, China Medical University, No.77 Puhe Road, Shenbei New District, Shenyang, 110122 China

**Keywords:** Meta-analysis, *CACNA1C*, rs1006737, rs2007044, rs4765905, Schizophrenia

## Abstract

**Background:**

The *CACNA1C* gene was defined as a risk gene for schizophrenia in a large genome-wide association study of European ancestry performed by the Psychiatric Genomics Consortium. Previous meta-analyses focused on the association between the *CACNA1C* gene rs1006737 and schizophrenia. The present study focused on whether there was an ancestral difference in the effect of the *CACNA1C* gene rs1006737 on schizophrenia. rs2007044 and rs4765905 were analyzed for their effect on the risk of schizophrenia.

**Methods:**

Pooled, subgroup, sensitivity, and publication bias analysis were conducted.

**Results:**

A total of 18 studies met the inclusion criteria, including fourteen rs1006737 studies (15,213 cases, 19,412 controls), three rs2007044 studies (6007 cases, 6518 controls), and two rs4765905 studies (2435 cases, 2639 controls). An allele model study also related rs2007044 and rs4765905 to schizophrenia. The overall meta-analysis for rs1006737, which included the allele contrast, dominant, recessive, codominance, and complete overdominance models, showed significant differences between rs1006737 and schizophrenia. However, the ancestral-based subgroup analysis for rs1006737 found that the genotypes GG and GG + GA were only protective factors for schizophrenia in Europeans. In contrast, the rs1006737 GA genotype only reduced the risk of schizophrenia in Asians.

**Conclusions:**

Rs1006737, rs2007044, and rs4765905 of the *CACNA1C* gene were associated with susceptibility to schizophrenia. However, the influence model for rs1006737 on schizophrenia in Asians and Europeans demonstrated both similarities and differences between the two ancestors.

## Background

Schizophrenia is a chronic, disabling brain disease characterized by delusions, hallucinations, and formal thought disorders in addition to a decline in socio-occupational functioning [1]. Studies with twins [2] and adoptive families [3] have shown that genetic factors are an important cause of schizophrenia. The L-type voltage-gated calcium channels play a unique role in behavioral extinction [4], inhibitory learning, and the maturation of adult cognitive function [5]. The two principal pore-forming subunits of these channels expressed in neurons are the α1C and α1D subtypes [6]. The α1C subtype is encoded by the *CACNA1C* gene, which is considered a risk factor for schizophrenia based on a large genome-wide association study (GWAS) of European ancestry performed by the Psychiatric Genomics Consortium (PGC) [7]. A growing body of research supports a key role for *CACNA1C* in schizophrenia in Europeans. Ivorra et al. [8] found that the rs1006737 polymorphism of the *CACNA1C* gene is strongly associated with schizophrenia and bipolar disorder in a Spanish sample. Wolf et al. [9] suggested that the *CACNA1C* genotype may explain inter-individual differences in the amygdala volume among patients with schizophrenia in the German sample. The amygdala is not only involved in associative learning but also regulates additional cognitive processes, such as memory and attention [10]. Fatima et al. [11] detected a significant difference in the genotype and allele frequencies for the rs4765905 polymorphism between patients and controls, confirming the hypothesis that the *CACNA1C* gene was associated with schizophrenia in the Pakistani sample. Rs1006737 and rs4765905 are located in intron 3 of *CACNA1C* gene. And previous study [12] have shown that disease-related SNPs in the *CACNA1C* gene (including rs1006737 and rs4765905) were proven to be expression quantitative trait loci (eQTLs), which are located in a region interacting with the promoter of *CACNA1C*, and may regulate the expression of *CACNA1C* in the brain.

Based on these findings, we were curious to see if the *CACNA1C* gene had the same effect on schizophrenia in Asians as it did in the Europeans. The meta-analysis of Zheng et al. [13] and Jiang et al. [14] showed that there was no heterogeneity between the *CACNA1C* rs1006737 polymorphism in East Asians and Europeans. He et al. [15] also showed that rs1006737 was associated with both schizophrenia and major depressive disorder in the Han Chinese sample. Additional rs1006737 meta-analysis showed an association between this *CACNA1C* polymorphism and schizophrenia in both the Europeans and Asians when the samples were stratified by ethnicity [16]. However, in a follow-up to the top European GWAS hits, The genotyping performed by Takahashi et al. [17] implicated loci in additional schizophrenia family samples from China and Japan and found no association between 12 polymorphisms (e.g., rs4765905 in the *CACNA1C* gene) and schizophrenia. Consistent with this finding, Hori et al. [18] found no significant difference in the genotype or allele frequency of the *CACNA1C* rs1006737 polymorphism between schizophrenia patients and controls in a Japanese sample.

In summary, there is no consensus on whether *CACNA1C* is associated with schizophrenia or if there are differences in susceptibility to schizophrenia between Asians and Europeans. Therefore, we performed an updated comprehensive meta-analysis on the relationship between *CACNA1C* gene polymorphisms and schizophrenia, which included case-control studies.

## Methods

### Literature search strategy

To identify eligible studies, we searched two electronic databases, PubMed and China’s National Knowledge Infrastructure [CNKI]. English studies were obtained by PubMed (2011-Present) database, and Chinese studies were obtained by CNKI (2013-Present) database. Only completed peer-review studies have the potential to be included in the present meta-analysis. The last search update was in November 2019. Rs1006737, rs2007044, rs4765905, *CACNA1C*, and schizophrenia were selected as search keywords.

The inclusion criteria for the present study were: a. including patients with schizophrenia; b. containing detailed genotypes and allele frequencies; c. including healthy control population; d. stating *CACNA1C* may be a potential gene of schizophrenia; e. the type of studies were case-control studies. The current exclusion criteria for meta-analysis were: a. no schizophrenic patients; b. no detailed genotype frequency data; c. no controls; d. abstracts, meta-analysis or reviews; e. not case-control studies; f. including repeated sample; g. containing 2014 PGC GWAS data [19].

### Data extraction

Two independent authors conduct data extraction according to the inclusion and exclusion criteria. If there was inconsistency between the two authors, they will hold discussions until an agreement was reached. Table 1 summarizes the first author’s last name, publication year, region, ancestry, source of control, mean age of control group, gender index, number of case group and control group, and the number of genotypes in case and control group.

**Table 1:** The main characteristics of the studies included in the meta-analysis

Author	Year	Country	Ancestor	Source of control group	Mean age of control group	Gender index (case)	Gender index (control)	Case/Control
**Rs1006737**								
Fatima [11]	2017	Pakistani	Caucasian	Population based	44	0.33	0.71	508/300
Lubeiro [20]	2018	Spain	Caucasian	Population based	29.52	0.72	0.98	50/101
Mallas [21]	2016	Mixed	Mixed	Population based	35.79	0.26	0.85	63/124
Porcelli [22]	2015	Korean	Asian	Hospital based	45.36	0.73	1.22	176/326
Ivorra [8]	2014	Spain	Caucasian	Mixed	43.61	0.79	0.75	3063/2847
He [15]	2013	China	Asian	Population based	30.6	0.53	0.86	1235/1235
Guan [23]	2013	China	Asian	Population based	34.2	0.87	0.83	1430/1570
Galaktionova [24]	2013	Russia	Caucasian	Population based	36	2.24	0.90	188/192
Zheng [13]	2013	China	Asian	Population based	32.4	1.05	1.04	5893/6319
Hori [18]	2012	Japan	Asian	Population based	46	0.82	1.93	552/1132
Zhang [25]	2011	China	Asian	Population based	22.3	0.49	0.60	318/401
Nyegaard [26]	2010	Denmark	Caucasian	Population based	–	–	–	976/1489
Bigos [27]	2010		Caucasian	Population based	33.09	0.230	1.16	282/440
Green [28]	2009	UK	Caucasian	Population based	–	0.47	1.04	479/2936
**Rs2007044**								
Bustillo [29]	2017	United States	Caucasian	Population based	36	0.26	0.37	53/129
Zhang [30]	2018	China	Asian	Hospital based	27.14	0.15	0.28	53/129
**Rs4765905**								
Sudesh [31]	2018	India	Indian	Population based	38.73	1.01	0.483	1005/1069

**Notes:** Gender index = female/male; Guan’s study included the main characteristics of both rs1006737 and rs4765905; Zheng’s study included the main characteristics of both rs1006737 and rs2007044

### Sensitivity analysis and publication bias

Sensitivity analysis was used to evaluate whether the combined results were stable and reliable. Funnel plots (the x-axis was the logarithm of OR, and the y-axis was the standard error of the logarithm of the OR) were used to determine whether the included studies had publication bias. Egger’s test [32] was used to assess the level of publication bias. *P*-value greater than 0.05 indicates a publication bias.

### Statistical analysis

We evaluated the Hardy-Weinberg equilibrium (HWE) in the control group of each study using Pearson’s chi-square test. The odds ratios (ORs) and 95% confidence intervals (CIs) were used to evaluate the correlation between polymorphisms rs1006737, rs4765905, rs2007044 and schizophrenia risk. The Cochran’s Q-test [33] and I^2^ statistics [34] were selected to check the heterogeneity among studies. Cochran’s Q-test is qualitative. If a *P*-value was greater than 0.1, it means a lack of heterogeneity, and the fixed effect model (Mantel-Haenszel) was selected. Conversely, a *p*-value was less than 0.1, indicating the existence of heterogeneity. The random effect model (M-H heterogeneity) was selected [35]. I^2^ is quantitative statistics, which refers to the ratio of the variation between studies to the total variation. It was divided into three groups according to heterogeneity level: low (less than 25%), moderate (25 to 75%), and high (greater than 75%). The “a” was marked as the risk allele. We use allelic: a vs. A, dominant: Aa + AA vs. AA, recessive: aa vs. AA, and codominant: aa vs. AA and Aa vs. AA, and complete overdominance: AA + aa vs. Aa models to calculate the pooled ORs. Besides, a subgroup analysis based on ancestry was conducted.

Meta-regression analysis was performed to assess the impact of different variables (mean age of control group and sex indexes) on the analysis. Statistical calculations were performed using Stata version 12.0 (StataCorp LP, College Station, TX, USA) software. *P*-value less than 0.05 indicates statistical difference (two tails).

## Results

**Fig. 1:**
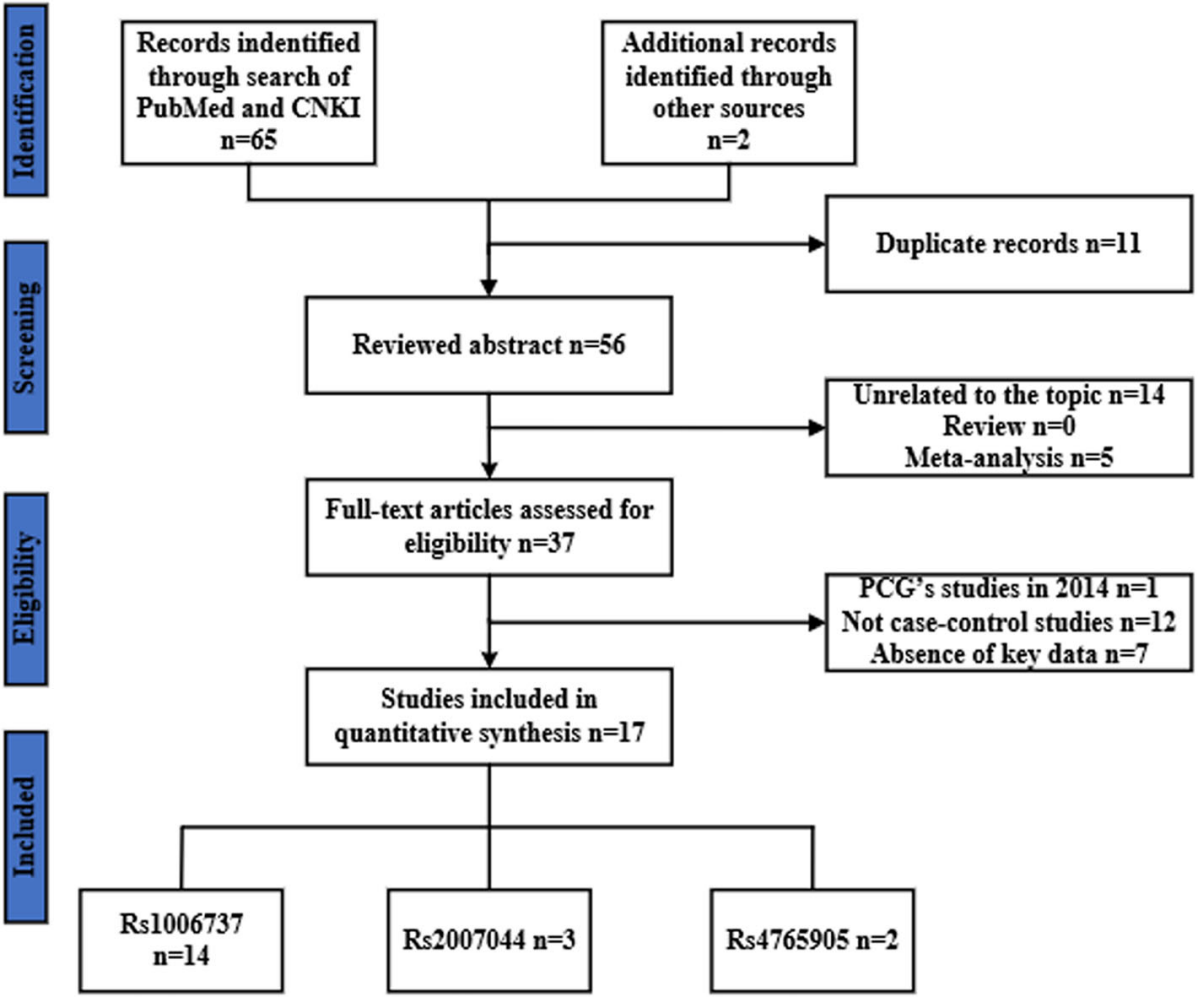
Flow diagram for the search and selection of the included studies. A total of 67 relevant English and Chinese studies were retrieved from the PubMed and CNKI databases. Following removal of the studies that did not meet our inclusion criteria, a total of 19 studies were included in the meta-analysis, including 14 rs1006737 studies, three rs2007044 studies, and two rs4765905 studies

We investigated 67 related articles from PubMed and CNKI electronic databases. Studies that did not conform to the inclusion criteria were excluded, and 18 studies were available for meta-analysis (Fig.1). Specifically, these 18 studies included 14 rs1006737 studies (15,213 cases and 19,412 controls), three rs2007044 studies (6007 cases and 6518 controls), and two rs4765905 studies (2435 cases and 2639 controls). The allelic and genotype distributions of all included studies were summarized in Table 2.

**Table 2:** The distributions of the allele frequency and genotype in the included studies

Author	Year	Genotype distribution						***P***HWE		Allele frequency		
ss		Cases, n			Controls, n				Cases (%)		Control (%)	
		AA	Aa	aa	AA	Aa	aa		A	a	A	a
**Rs1006737**												
Lubeiro	2018	25	23	2	58	38	5	0.70	73 (73.0)	27 (27.0)	154 (76.2)	48 (23.8)
Fatima	2017	393	84	17	235	54	9	0.01	870 (88.1)	118 (11.9)	524 (88.0)	72 (12.0)
Ivorra	2014	1417	1271	293	1420	1124	240	0.41	4105 (68.9)	1857 (31.1)	3964 (71.2)	1604 (28.8)
Galaktionova	2013	78	85	23	80	90	22	0.66	241 (64.8)	131 (35.2)	250 (65.1)	134 (34.9)
Nyegaard	2010	402	444	130	656	675	158	0.42	1248 (63.9)	704 (36.1)	1987 (66.7)	991 (33.3)
Bigos	2010	120	115	47	191	205	44	0.31	355 (62.9)	209 (37.1)	587 (66.7)	293 (33.3)
Green	2009	205	208	66	1367	1233	336	0.02	618 (64.5)	340 (35.5)	3967 (67.6)	1095 (32.4)
Mallas	2016	23	30	10	56	51	17	0.33	76 (60.3)	50 (39.7)	163 (65.7)	85 (34.3)
Porcelli	2015	153	23	0	301	23	2	0.11	329 (93.5)	23 (6.5)	625 (95.9)	27 (4.1)
He	2013	996	220	14	1053	166	9	0.39	2212 (89.9)	248 (10.1)	2272 (92.5)	184 (7.5)
Guan	2013	1061	343	26	1223	327	20	0.72	2465 (86.2)	395 (13.8)	2773 (88.3)	367 (11.7)
Zheng	2013	5239	635	19	5706	597	16	0.93	11,113 (94.3)	673 (5.7)	12,009 (95.0)	629 (5.0)
Hori	2012	480	70	2	1002	127	3	0.63	1030 (93.3)	74 (6.7)	2131 (94.1)	133 (5.9)
Zhang	2011	280	37	1	357	42	2	0.53	597 (93.9)	39 (6.1)	756 (94.3)	46 (5.7)
**Rs2007044**												
Zhang	2018	24	25	4	58	57	14	1.00	73 (68.9)	33 (31.1)	173 (67.1)	85 (32.9)
Bustillo	2017	26	23	9	35	21	11	0.02	75 (64.7)	41 (35.3)	91 (67.9)	43 (32.1)
Zheng	2014	2797	2540	559	3166	2597	559	0.42	8134 (77.5)	3658 (22.5)	8929 (70.7)	3715 (29.3)
**Rs4765905**												
Sudesh	2018	579	307	51	668	286	38	0.29	1465 (78.2)	409 (21.8)	1622 (81.8)	362 (18.2)
Guan	2013	1307	360	33	1195	352	24	0.74	2434 (71.6)	426 (28.4)	2741 (87.2)	399 (12.8)

**Notes:***P*_HWE_, *P*-value of the Hardy–Weinberg equilibrium; A, wild-type allele; a, mutant allele

### *CACNA1C* Rs1006737 polymorphism

ORs were estimated in allelic (A vs. G), dominant (GA + AA vs. GG), recessive (AA vs. GG), codominance (AA vs. GG and GA vs. GG), and complete overdominance (GG + AA vs. GA) models (A was the risk allele). All models except for the codominance model (GA vs. GG) were performed using the fixed effects model (M-H) due to the low heterogeneity. In contrast, the codominance model (GA vs. GG) was performed using the random effects model (M-H) due to its high heterogeneity (I^2^ = 99%).

The overall meta-analysis (Table 3) showed that rs1006737 was significantly associated with schizophrenia risk in the following models: allele model (A vs. G: *P* = 0.000, OR = 1.151, 95% CI = 1.100–1.204, I^2^ = 0.0%, *P* heterogeneity = 0.867); dominant model (GA + AA vs. GG: *P* = 0.000, OR = 1.169, 95% CI = 1.107–1.234, I^2^ = 0.0%, *P* heterogeneity = 0.786); recessive model (AA vs. GG + GA: *P* = 0.001, OR = 1.215, 95% CI = 1.085–1.360, I^2^ = 0.0%, *P* heterogeneity = 0.999); codominance models (AA vs. GG: *P* = 0.000, OR = 1.296, 95% CI = 1.151–1.459, I^2^ = 0.0%, *P* heterogeneity = 0.993); codominance model (GA vs. GG: *P* = 0.000, OR = 0.064, 95% CI = 0.024–0.169, I^2^ = 99%, *P* heterogeneity = 0.000), and complete overdominance model (GG + AA vs. GA: *P* = 0.000, OR = 0.897, 95% CI = 0.849–0.948, I^2^ = 26.1%, *P* heterogeneity = 0.173).

**Table 3:** The main results of the overall meta-analysis of *CACNA1C* polymorphisms

Genetic model	OR	95% CI	***P***-value	I^**2**^(%)	***P***_**h**_	Combination method
**Rs1006737**						
Allele contrast	1.151	1.100–1.204	**0.000**	0.0	0.867	Fixed effects model
Dominant	1.169	1.107–1.234	**0.000**	0.0	0.786	Fixed effects model
Recessive	1.215	1.085–1.360	**0.001**	0.0	0.999	Fixed effects model
Codominance AA vs. GG	1.296	1.151–1.459	**0.000**	0.0	0.993	Fixed effects model
Codominance GA vs. GG	0.064	0.024–0.169	**0.000**	99.0	**0.000**	Random effects model
Complete overdominance	0.897	0.849–0.948	**0.000**	26.1	0.173	Fixed effects model
**Rs2007044**						
Allele contrast	1.080	1.023–1.139	**0.006**	0.0	0.785	Fixed effects model
**Rs4765905**						
Allele contrast	1.225	1.100–1.364	**0.000**	0.0	0.719	Fixed effects model

**Notes:** I^2^ represents the variation in OR attributable to heterogeneity. *P*_h_ represents the *P*-value of the Q-test for heterogeneity

**Abbreviations:***CI* confidence interval; *OR* odds ratio

Subsequently, subgroup analysis based on ancestor was performed for rs1006737. For the Caucasians, there were seven studies that included a total of 5546 patients with schizophrenia and 8305 controls. Rs1006737 was associated with schizophrenia using all but one genetic model (A vs. G, *P* = 0.000, OR = 1.121, 95% CI = 1.060–1.186; GA + AA vs. GG, *P* = 0.001, OR = 1.127, 95% CI = 1.047–1.213; AA vs. GG + GA, *P* = 0.003, OR = 1.203, 95% CI = 1.067–1.357; AA vs. GG, *P* = 0.000, OR = 1.284, 95% CI = 1.131–1.456; GA vs. GG, *P* = 0.001, OR = 0.279, 95% CI = 0.132–0.587). The complete overdominance model (GG + AA vs. GA) resulted in an OR = 0.959, 95% CI = 0.891–1.033, and *P* = 0.272.

The subgroup analysis also included six studies involving Asians with a total of 9604 patients with schizophrenia and 10,983 controls. Rs1006737 was associated with schizophrenia using the following models: allele contrast model (A vs. G: *P* = 0.000, OR = 1.206, 95% CI = 1.117–1.303); dominant model (GA + AA vs. GG: *P* = 0.000, OR = 1.219, 95% CI = 1.123–1.323); codominance model (GA vs. GG; OR = 0.008, 95% CI = 0.004–0.017, *P* = 0.000); complete overdominance model (GG + AA vs. GA: *P* = 0.000, OR = 0.827, 95% CI = 0.761–0.899). There was no association observed using the recessive (AA vs. GG + GA: *P* = 0.125, OR = 1.336, 95% CI = 0.922–1.936) or codominance model (AA vs. GG: *P* = 0.086, OR = 1.384, 95% CI = 0.955–2.006) models (Table 4). Neither the mean age of the control group (slope = 0.995, 95% CI = 0.985–1.005, *P* = 0.265) nor the sex indexes (case group, slope = 0.943, 95% CI = 0.797–1.115, *P* = 0.455; control group, slope = 1.059, 95% CI = 0.829–1.352, *P* = 0.616) had any significant impact on the results.

**Table 4:** Subgroup analysis of the association between rs1006737 and the risk of schizophrenia

Ancestor	Summary of pooled ORs			Heterogeneity test	
	OR	95% CI	***P***-value	I^**2**^(%)	***P***_**h**_
**Asian**					
Allele contrast	1.206	1.117–1.303	**0.000**	0.0	0.583
Dominant	1.219	1.123–1.323	**0.000**	0.0	0.484
Recessive	1.336	0.922–1.936	0.125	0.0	0.939
Codominance AA vs. GG	1.384	0.955–2.006	0.086	0.0	0.932
Codominance GA vs. GG	0.008	0.004–0.017	**0.000**	83.6	**0.000**
Complete overdominance	0.827	0.761–0.899	**0.000**	0.0	0.434
**Caucasian**					
Allele contrast	1.121	1.060–1.186	**0.000**	0.0	0.964
Dominant	1.127	1.047–1.213	**0.001**	0.0	0.919
Recessive	1.203	1.067–1.357	**0.003**	0.0	0.987
Codominance AA vs. GG	1.284	1.131–1.456	**0.000**	0.0	0.893
Codominance GA vs. GG	0.279	0.132–0.587	**0.001**	98.0	**0.000**
Complete overdominance	0.959	0.891–1.033	0.272	0.0	0.457

**Notes:** I^2^ represents the variation in OR attributable to heterogeneity. *P*_h_ represents the *P*-value of the Q-test for heterogeneity **Abbreviations:***CI* confidence interval; *OR* odds ratio

### Rs2007044 and rs4765905 polymorphisms of *CACNA1C*

Allele G of rs2007044 and allele C of rs4765905 were defined as risk alleles. Because relatively few studies related to rs2007044 and rs4765905 were included in the meta-analysis, only the allele model for these two polymorphisms was analyzed. Significant differences between the patients and controls were observed for both rs2007044 (G vs. A: *P* = 0.006, OR = 1.080, 95% CI = 1.023–1.139) and rs4765905 (C vs. G: *P* = 0.000, OR = 1.225, 95% CI = 1.100–1.364). The main results are presented in Table 3.

### Sensitivity analysis and publication bias

**Fig. 2:**
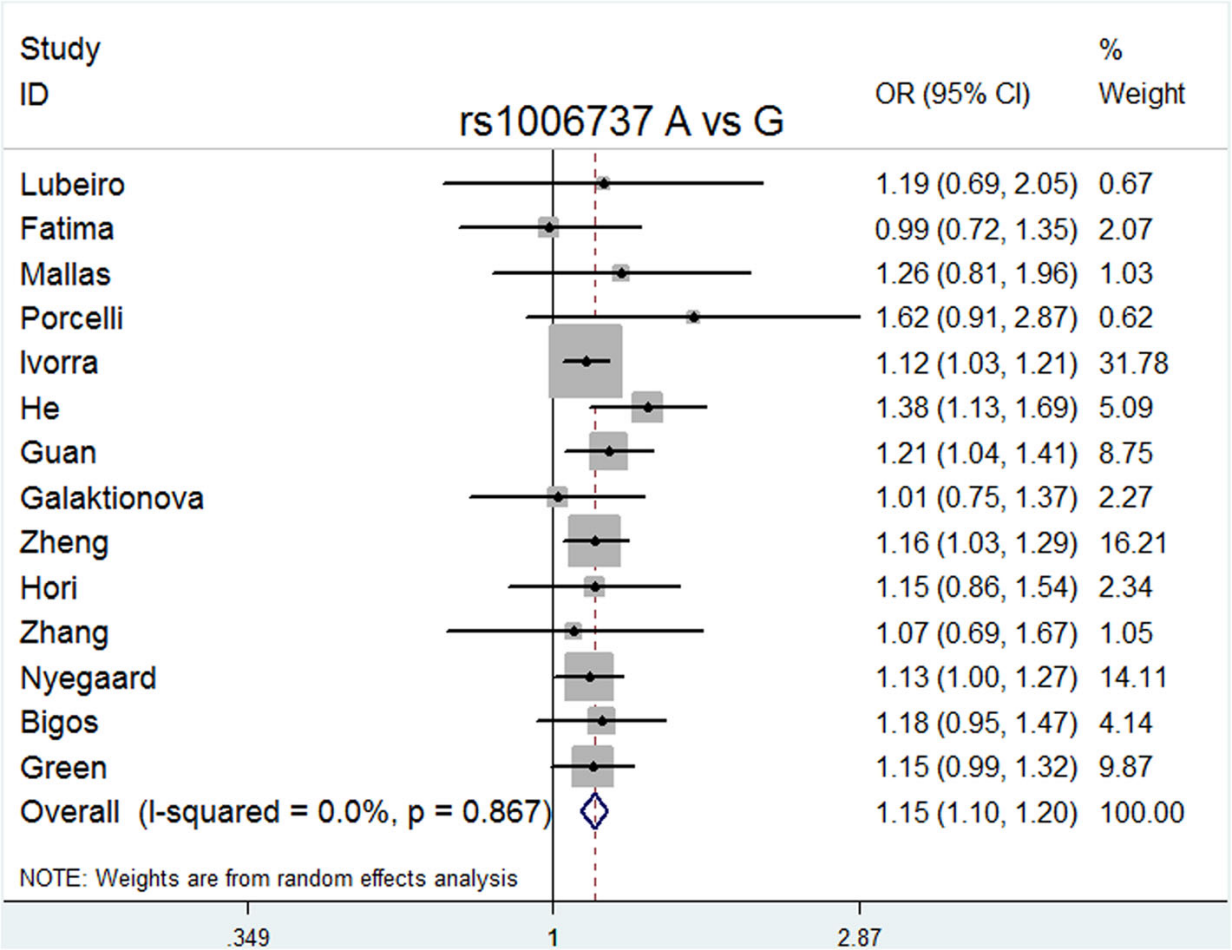
Forest plot of the allele contrast model (A vs. G) for rs1006737. Significant differences between rs1006737 and the risk of schizophrenia were observed with the allele contrast model (T vs. G) (OR = 1.151, 95% CI = 1.100–1.204, *P* heterogeneity = 0.867, *P* = 0.000)

**Fig. 3:**
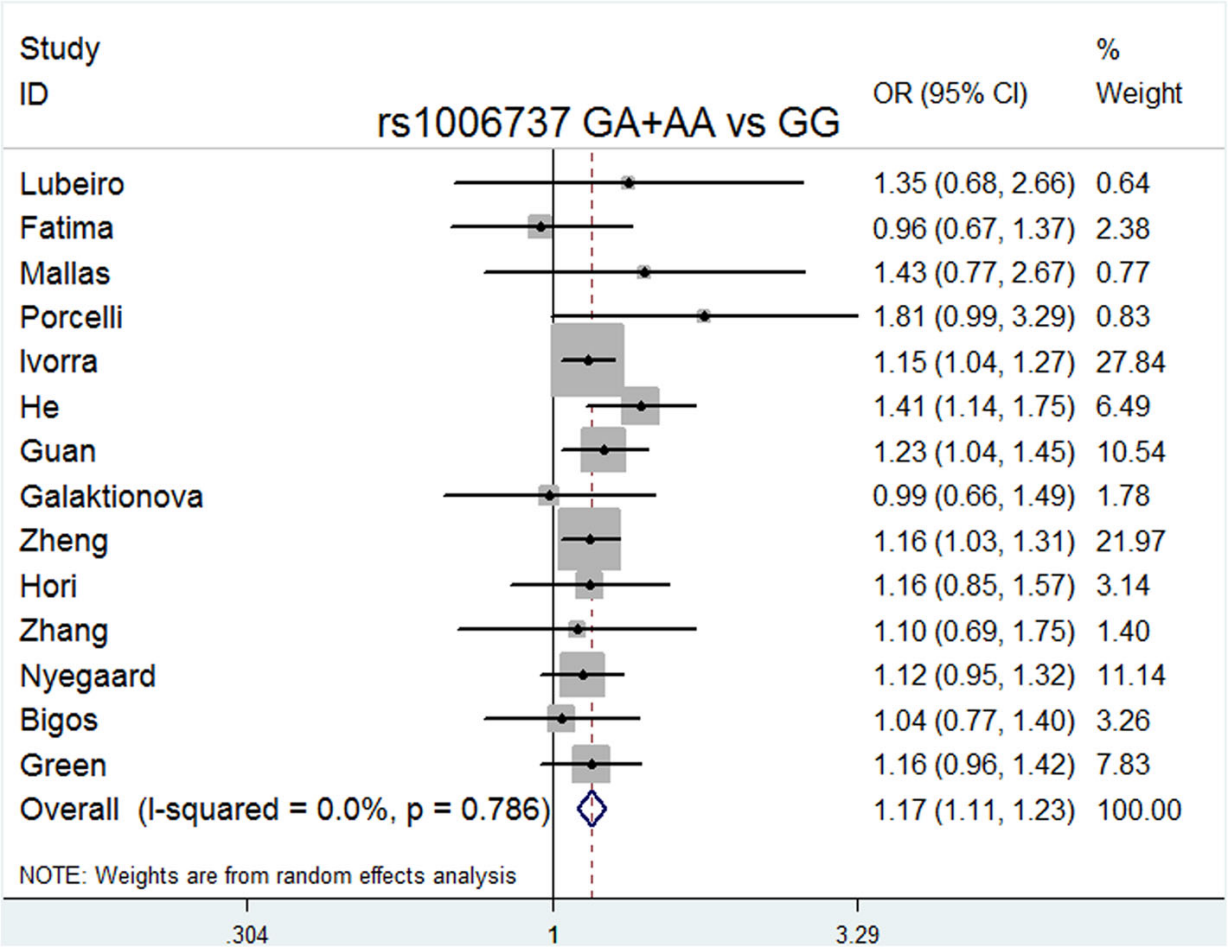
Forest plot of the dominant model (GA + AA vs. GG) for rs1006737. Significant differences between rs1006737 and the risk of schizophrenia were observed with the dominant model (GA + AA vs. GG) (OR = 1.169, 95% CI = 1.107–1.234, *P* heterogeneity = 0.786, *P* = 0.000)

**Fig. 4:**
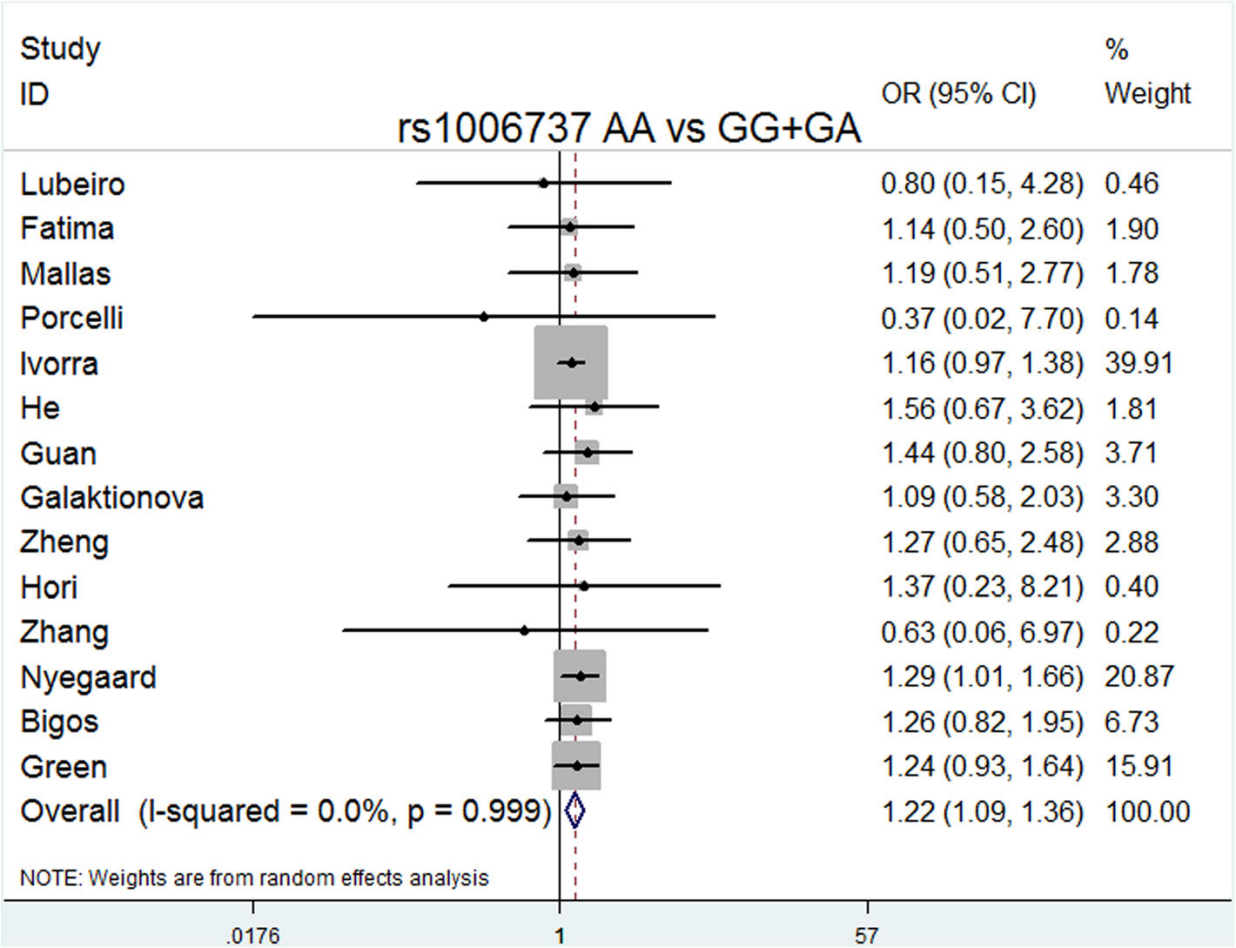
Forest plot of the recessive model (AA vs. GG + GA) for rs1006737. Significant differences between rs1006737 and the risk of schizophrenia were observed with the recessive model (AA vs. GG + GA) (OR = 1.215, 95% CI = 1.085–1.360, *P* heterogeneity = 0.999, *P* = 0.001)

**Fig. 5:**
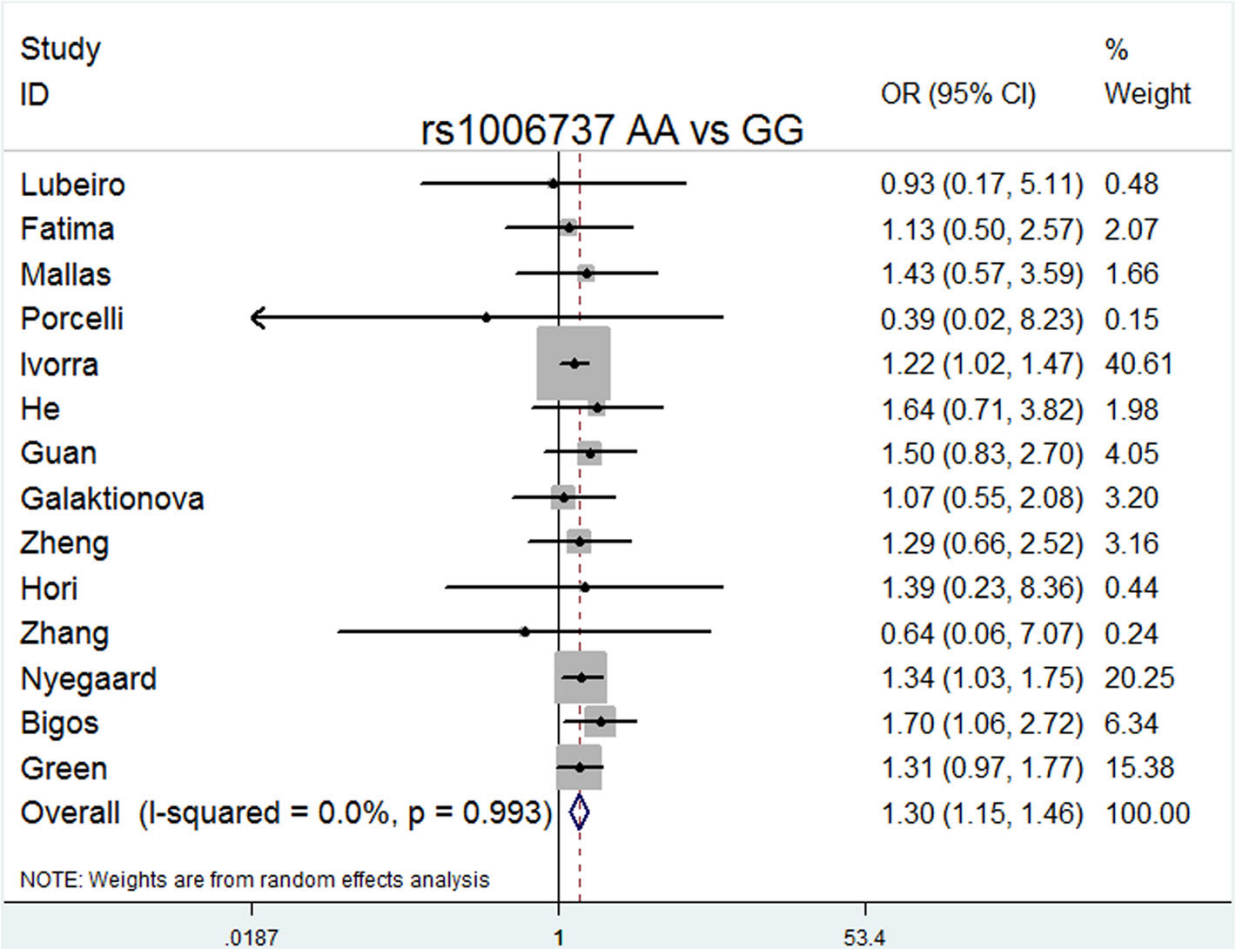
Forest plot of the complete codominance model (AA vs. GG) for rs1006737. Significant differences between rs1006737 and the risk of schizophrenia were observed with the codominance model (AA vs. GG) (OR = 1.296, 95% CI = 1.151–1.459, *P* heterogeneity = 0.993, *P* = 0.000)

**Fig. 6:**
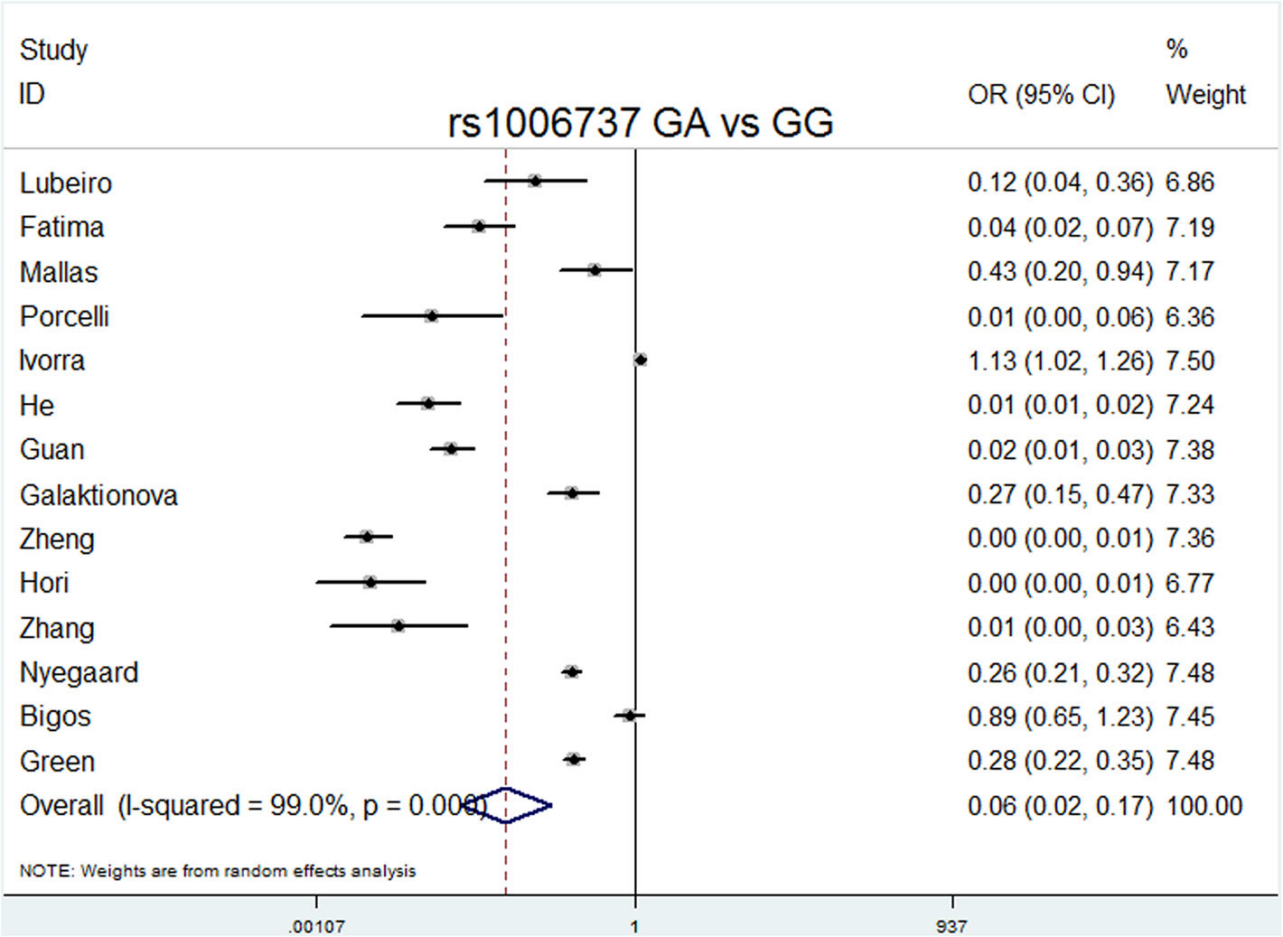
Forest plot of the codominance model (GA vs. GG) for rs1006737. Significant differences between rs1006737 and the risk of schizophrenia were observed with the codominance model (GA vs. GG) (OR = 0.064, 95% CI = 0.024–0.169, *P* heterogeneity = 0.000, *P* = 0.000)

**Fig. 7:**
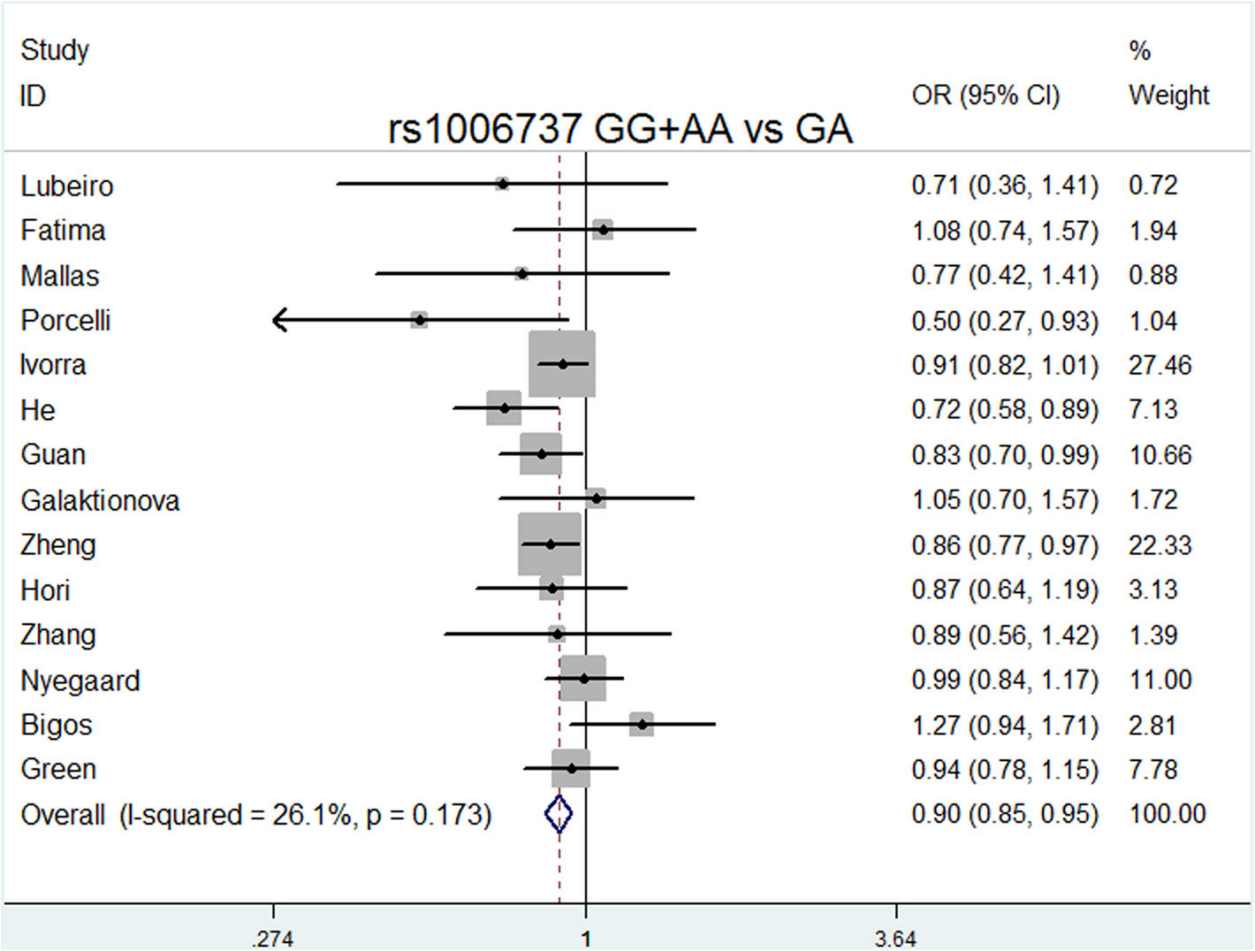
Forest plot of the complete overdominance model (GG + AA vs. GA) for rs1006737. Significant differences between rs1006737 and the risk of schizophrenia were observed with the complete overdominance model (GG + AA vs. GA) (OR = 0.897, 95% CI = 0.849–0.948, *P* heterogeneity = 0.173, *P* = 0.000)

**Fig. 8:**
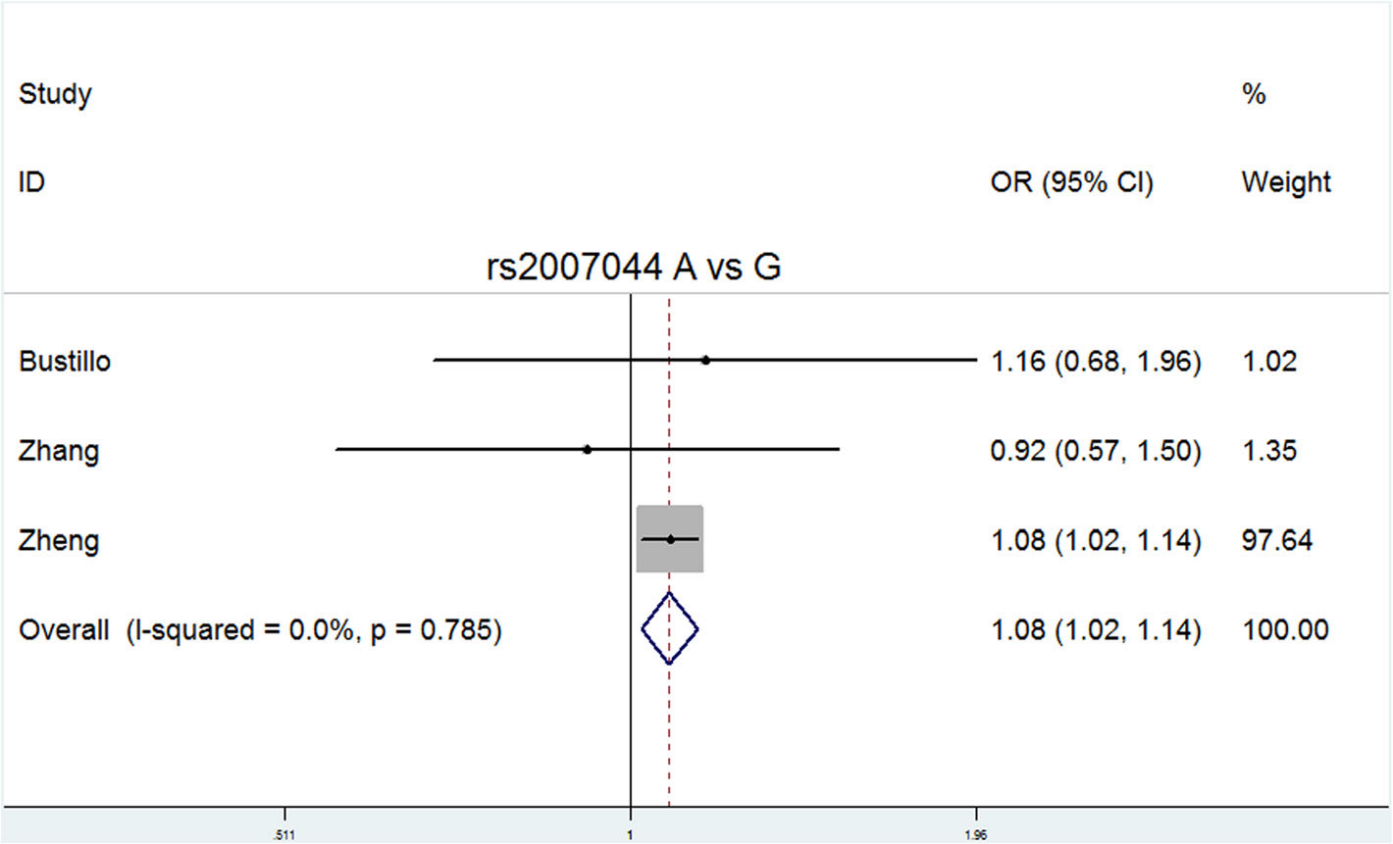
Forest plot of the allele contrast model (A vs. G) for rs2007044. Significant differences between rs2007044 and the risk of schizophrenia were observed with the allele contrast model (A vs. G) (OR = 1.080, 95% CI = 1.023–1.139, *P* heterogeneity = 0.785, *P* = 0.006)

**Fig. 9:**
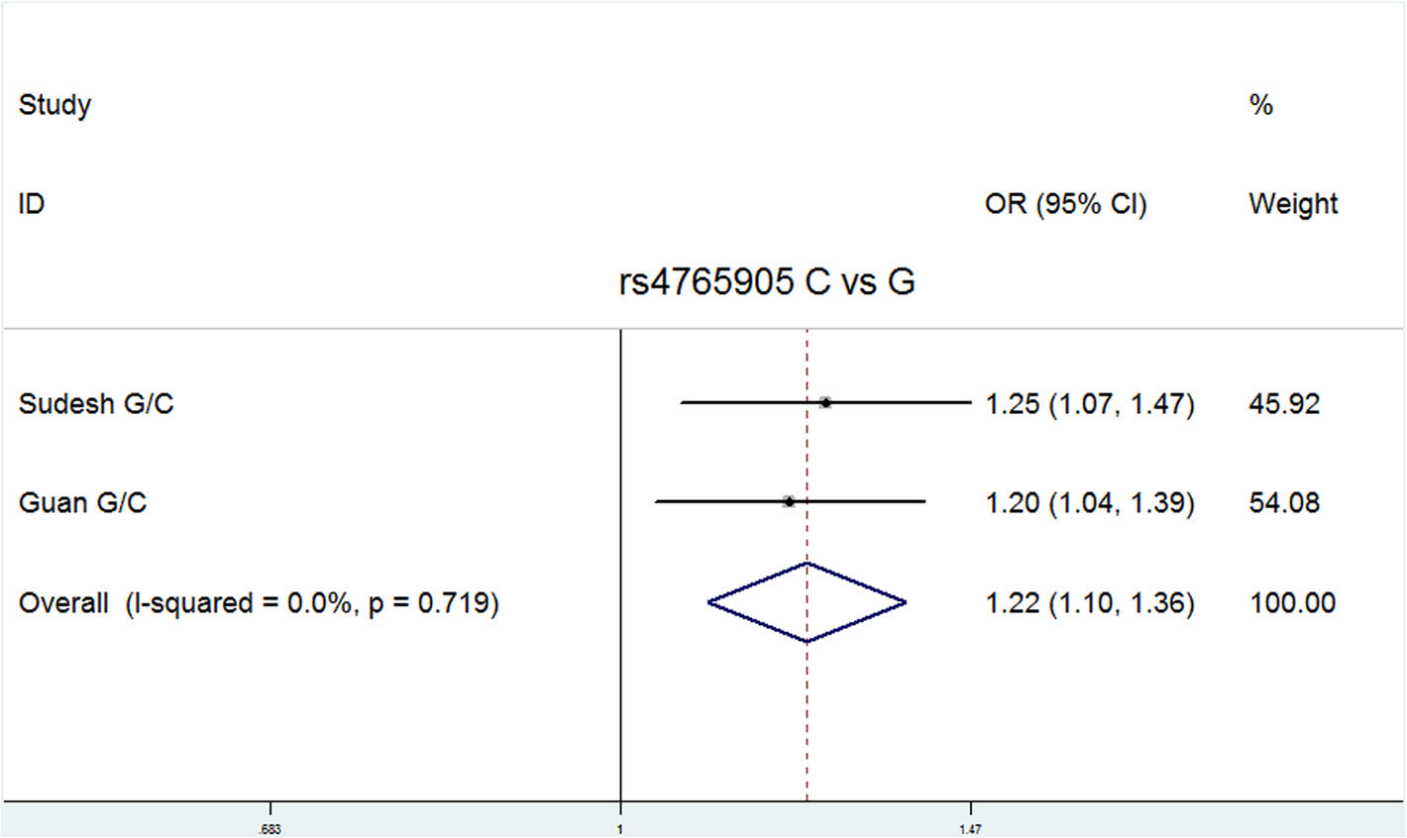
Forest plot of the allele contrast model (C vs. G) for rs4765905. Significant differences between rs4765905 and the risk of schizophrenia were observed with the allele contrast model (C vs. G) (OR = 1.225, 95% CI = 1.100–1.364, *P* heterogeneity = 0.719, *P* = 0.000)

Delete each study item by item, and then calculate the significance of the new meta-analysis results consisting of the remaining studies [36]. We did not observe statistically significant differences, indicating that the current results are reliable and stable and have not been affected by any separate studies. (Table 5). The symmetry of the funnel plots can reflect the publication bias (Figs. 2, 3, 4, 5, 6, 7, 8 and 9). Egger’s test quantifies the publication bias analysis. Due to the lack of studies on rs4765905, the efficacy of the Egger’s test was limited, and the symmetry of the funnel plot could not be detected. We did not find publication bias in the rs2007044 (G vs. A; *t* = − 0.43, *P* = 0.743) or rs4765905 (A vs. G; *t* = 0.86, *P* = 0.407) allele model. For rs1006737, there were no publication biases in the dominant (GA + AA vs. GG: *P* = 0.613, *t* = 0.52), recessive (TT vs. GG + GT: *P* = 0.507, *t* = − 0.68), codominant (AA vs. GG: *P* = 0.713, *t* = − 0.38) and complete overdominance (GG + TT vs. GT: *P* = 0.762, *t* = − 0.31) models. However, there was a publication bias for the rs1006737 polymorphism with the codominance model (AA vs. GG; *t* = − 3.88, *P* = 0.002).

**Table 5:** Results of the sensitivity analysis for the *CACNA1C* rs1006737 polymorphism

Excluded		OR	95% CI	***P***-value	***P***_**h**_
Study	Sample				
Lubeiro	Caucasian	1.1506206	1.0998443–1.2037411	0.000	0.815
Fatima	Caucasian	1.1546086	1.1033095–1.2082929	0.000	0.878
Mallas	Mixed	1.1497633	1.0989355–1.202942	0.000	0.826
Porcelli	Asian	1.1485037	1.0978361–1.2015097	0.000	0.903
Ivorra	Caucasian	1.166579	1.1047648–1.2318518	0.000	0.866
He	Asian	1.1393697	1.0879437–1.1932266	0.000	0.981
Guan	Asian	1.1452636	1.0925845–1.2004827	0.000	0.847
Galaktionova	Caucasian	1.1542471	1.1029066–1.2079774	0.000	0.863
Zheng	Asian	1.1498204	1.094684–1.2077338	0.000	0.815
Hori	Asian	1.1508508	1.0996418–1.2044445	0.000	0.814
Zhang	Asian	1.1517017	1.1007836–1.204975	0.000	0.821
Nyegaard	Caucasian	1.154137	1.0994588–1.2115344	0.000	0.821
Bigos	Caucasian	1.1496404	1.0980188–1.2036888	0.000	0.818
Green	Caucasian	1.151419	1.0981367–1.2072866	0.000	0.814

**Notes:***P*_h_ represents the *P*-value of the Q-test for heterogeneity

**Abbreviations:***CI* confidence interval; *OR* odds ratio

## Discussion

*CACNA1C* is associated with bipolar disorder [37], autism spectrum disorder [38], major depression [15], and other central nervous system (CNS) disorders [39]. However, the association between the *CACNA1C* gene and schizophrenia has not been determined. It is also unclear whether the *CACNA1C* gene has the same effect on schizophrenia in both Asians and Europeans. Therefore, we conducted a comprehensive meta-analysis on the association between the *CACNA1C* rs1006737, rs2007044, and rs4765905 polymorphisms and schizophrenia. In the overall analysis, rs1006737 was associated with the risk of schizophrenia in all five genetic models, and rs2007044 and rs4765905 were also related to schizophrenia in the allele model, implying that the *CACNA1C* gene may influence the risk of schizophrenia. This view is consistent with the results of previous meta-analyses [13, 14, 16, 35, 40, 41].

When we conducted a race-based subgroup analysis of rs1006737, we found that the effects of rs1006737 on schizophrenia in the Asians and Europeans had both similarities and differences. According to the results obtained with the allele (A vs. G) and dominant (GA + AA vs. GG) models, the effect of rs1006737 on the risk of schizophrenia in the Europeans and Asians was consistent (i.e., allele A and genotype GA + AA were protective factors against the development of schizophrenia). However, analysis by the recessive (AA vs. GG + GA) and codominant (AA vs. GG) models showed that the genotype GG + GA was only a risk factor for schizophrenia in the European sample. In contrast, according to the complete overdominance model (GG + AA vs. GA), the GA genotype of rs1006737 only reduced the risk of schizophrenia in the Asian sample. These data suggest that the effect of rs1006737 on schizophrenia is ancestrally diverse.

The current study has two limitations. Due to significant heterogeneity (I^2^ = 99.0%) and publication bias (Egger’s test *P* = 0.002), the codominant model (GA vs. GG) was not reliable and, therefore, was not a valid gene model for evaluating the rs1006737 polymorphism. In addition, there were few studies on the association between rs2007044 or rs4765905 and schizophrenia. Thus, additional high-quality studies are needed to support our analysis.

This meta-analysis study advanced our understanding of the relationship between *CACNA1C* polymorphisms and schizophrenia compared to previous literature. First, the current study included more comprehensive studies. A recent meta-analysis of the *CACNA1C* gene and schizophrenia [40] contained nine studies on the association between rs1006737 and schizophrenia. In comparison, the current study included 14 studies on the association between this polymorphism and schizophrenia, including eight articles [11, 13, 15, 18, 23, 25, 26, 28] shared with [40] along with six additional studies [8, 20–22, 24, 27]. Second, compared to most of the meta-analysis on *CACNA1C* and schizophrenia, the current study not only included studies on rs1006737 and schizophrenia but also studies on the association between two other *CACNA1C* polymorphisms (rs2007044 and rs4765905) and schizophrenia. Although the study of Xiao et al. [41] also included these three polymorphisms, it only included samples from Asian samples. Because the current study included samples from both Asians and Europeans, it used a richer source of samples for the analysis. Finally, the current study focused on comparing the impact of rs1006737 on schizophrenia in Asian and European samples. Based on this analysis, the influence model of rs1006737 on schizophrenia in Asian and European samples identified both similarities and differences between the two samples.

## Conclusions

The *CACNA1C* rs1006737, rs2007044, and rs4765905 gene polymorphisms were associated with the susceptibility to schizophrenia. However, the influence model for rs1006737 on schizophrenia in Asians and Europeans demonstrated both similarities and differences between the two ancestors.

## Data Availability

All data generated or analyzed during this study are included in this published article.

## Abbreviations

GWAS: Genome-wide association study
SNP: Single nucleotide polymorphism
PGC: Psychiatric Genomics Consortium
OR: Odds ratio
95% CI: 95% confidence interval
